# A Narrative Review of Cancer-Related Fatigue (CRF) and Its Possible Pathogenesis

**DOI:** 10.3390/cells8070738

**Published:** 2019-07-18

**Authors:** Songwei Yang, Shifeng Chu, Yan Gao, Qidi Ai, Yingjiao Liu, Xun Li, Naihong Chen

**Affiliations:** 1 College of Pharmacy, Hunan University of Chinese Medicine, Changsha 410208, Hunan, China; 2 Hunan Engineering Technology Center of Standardization and Function of Chinese Herbal Decoction Pieces, Changsha 410208, Hunan, China; 3 State Key Laboratory of Bioactive Substances and Functions of Natural Medicines, Institute of Materia Medical, Neuroscience Center, Chinese Academy of Medical Sciences and Peking Union Medical College, Beijing 100050, China

**Keywords:** cancer-related fatigue, peripheral immune activation, skeletal muscle, mitochondrion, inflammatory cytokines, central nervous system

## Abstract

Many cancer patients suffer from severe fatigue when treated with chemotherapy or radiotherapy; however, the etiology and pathogenesis of this kind of fatigue remains unknown. Fatigue is associated with cancer itself, as well as adjuvant therapies and can persist for a long time. Cancer patients present a high degree of fatigue, which dramatically affects the quality of their everyday life. There are various clinical research studies and reviews that aimed to explore the mechanisms of cancer-related fatigue (CRF). However, there are certain limitations in these studies: For example, some studies have only blood biochemical texts without histopathological examination, and there has been insufficient systemic evaluation of the dynamic changes in relevant indexes. Thus, we present this narrative review to summarize previous studies on CRF and explore promising research directions. Plenty of evidence suggests a possible association between CRF and physiological dysfunction, including skeletal muscular and mitochondrial dysfunction, peripheral immune activation and inflammation dysfunction, as well as central nervous system (CNS) disorder. Mitochondrial DNA (mtDNA), mitochondrial structure, oxidative pressure, and some active factors such as ATP play significant roles that lead to the induction of CRF. Meanwhile, several pro-inflammatory and anti-inflammatory cytokines in the peripheral system, even in the CNS, significantly contribute to the occurrence of CRF. Moreover, CNS function disorders, such as neuropeptide, neurotransmitter, and hypothalamic-pituitary-adrenal (HPA) axis dysfunction, tend to amplify the sense of fatigue in cancer patients through various signaling pathways. There have been few accurate animal models established to further explore the molecular mechanisms of CRF due to different types of cancer, adjuvant therapy schedules, living environments, and physical status. It is imperative to develop appropriate animal models that can mimic human CRF and to explore additional mechanisms using histopathological and biochemical methods. Therefore, the main purpose of this review is to analyze the possible pathogenesis of CRF and recommend future research that will clarify CRF pathogenesis and facilitate the formulation of new treatment options.

## 1. Background

Fatigue is both a symptom and measurable sign of many diseases, but adequate criteria for the identification of fatigue are lacking. According to the CDC, fatigue is defined by several characteristics: (1) It happens gradually, (2) it can be mitigated by rest, (3) it lasts more than six months [1]. Moreover, fatigue ought to be distinguished from mental fatigue, which is associated with cognitive function disorder and muscle weakness [2]. Sleep studies have revealed that mental fatigue is different from somnolence and is interrelated with sleep disturbances and depression [3]. Muscular fatigue, which often occurs following exhaustive muscular exercise, is caused by the disordered electrophysiological rhythm of muscle relaxation and contraction [4]. Although they are distinct conditions, it is difficult to discriminate between mental and muscular fatigue since mental fatigue can occur with physical effects that are also present in muscular fatigue.

However, with increasing cancer morbidity as well as viable treatments, a different type of fatigue has become regularly recognized as a condition associated with cancer or cancer treatment, namely, cancer-related fatigue (CRF). CRF has been defined as: ”a distressing, persistent, subjective sense of tiredness or exhaustion related to cancer or cancer treatment which is not proportional to recent activity and interferes with usual functioning” [5]. Most cancer survivors are present with severe fatigue after the completion of cancer treatment, despite the lack of any signs of cancer recurrence, indicating that chemotherapy or radiotherapy promotes CRF [6,7]. A cross-sectional population-based study found that 39% of survivors of colorectal cancer (CRC) reported extreme fatigue compared with 22% of the healthy population, and a longitudinal study reported a CRF rate of 10% before therapy and 26% after therapy [8,9]. Thus, severe fatigue seems to occur at higher rates after adjuvant chemotherapy or radiotherapy. Putative mechanisms leading to CRF include cytokines, the hypothalamic-pituitary-adrenal (HPA) axis, autonomic nervous system function, insulin signaling, neurotransmitters, anemia, and psychosocial or medical factors [10]. To date, the underlying molecular mechanisms and treatment-related hazards that lead to CRF remain unknown [11]. To reveal the current knowledge of the molecular mechanisms that induce CRF, and explore directions of further research, we review the existing literature on CRF.

## 2. Prevalence of CRF and Its Association with Cancer and Cancer Therapy

CRF is a symptom of cancer cachexia, which is triggered by cancer or cancer therapies. Cancer cachexia significantly influences the patient’s quality of life and decreases survival rates [12,13,14]. In 2014, the prevalence of cancer cachexia in cancer patients was estimated to be 50%–80% and accounted for 20% of cancer-related deaths [15]. The main symptoms of cachexia include the decrease of body mass index (BMI) on the account of reduction of skeletal muscle mass, and can’t be alleviated by conventional nutritional support, which naturally induce extremely serious fatigue in cancer patients [16,17]. It was reported that cancer patients always experienced highest levels of fatigue at the end of cancer treatment, meanwhile some female patients felt equivalent severe fatigue throughout their treatment trajectory [18]. Previous studies indicated that CRF was a distressing symptom for female patients with stage I and II breast cancer, and cancer itself or cancer treatment can cause unremitting fatigue [19].

**Figure 1:**
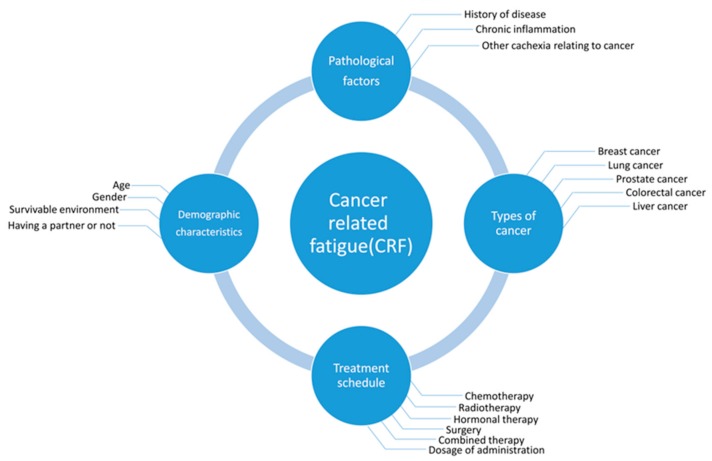
Cancer-related fatigue (CRF) is associated with various risk factors. The predominant factors are demographic characteristics, pathological factors, the types of cancer, and anti-cancer treatment schedules.

The pathogenic factors of severe fatigue include greater disease severity, radiotherapy, chemotherapy, surgery, hormone therapy, and a combination of these therapies, as well as demographic, lifestyle, and treatment characteristics. Breast cancer (BC) survivors with a partner were found to have a lower risk of developing severe fatigue, but the risk increased with advanced stages of BC; chemotherapy; a combination of surgery, chemotherapy, and radiotherapy; and hormone therapy combined with these treatments. The prevalence rate of severe fatigue ranges from 7% to 52%, and fatigue severity scores vary with different types of chemotherapy drugs or drug combinations [20] (Figure 1). It was reported that patients who received combinations of Cytoxan (cyclophosphamide), fluorouracil, Adriamycin (Doxorubicin) and/or Taxotere (docetaxel) experienced more severe fatigue than those who received only Taxol (paclitaxel) [21]. However, patients who received dose-dense or standard-dose of taxane treatments presented no significant difference in fatigue scores, indicating that fatigue severity changed significantly over time with different chemotherapy treatment types and a combination of strategies [22].

## 3. Skeletal Muscular and Mitochondrial Dysfunction

Once the structure and function of mitochondria are disrupted, the energy supply of cells decrease, leading to various unpleasant conditions, such as fatigue, muscle wasting, impaired regenerative tolerance, reduced exercise capacity, pain, and severe neurological diseases that are associated with anti-cancer chemotherapy [23,24]. Research on chemotherapy-receiving patients reported that chemotherapy always nonspecifically targets skeletal musculature, especially the mitochondria, inducing adverse side effects due to low energy supply and high oxidative stress [25]. Chemotherapy treatments cause long-term side effects in skeletal muscle that are different from those induced by the progression of cancer itself. Several mechanisms have been proposed to explain these effects: (1) DNA impairment that inhibits protein biosynthesis and transcription processes [26,27]; (2) generation of free radicals and oxidative products, which induce cellular damage, apoptosis, and necrosis [28,29]; (3) inhibited production of the topoisomerase II, which is important for nuclear DNA (nDNA) transcription [30]; and (4) activation and progression of intrinsic mitochondrial apoptosis. Moreover, mtDNA seems to be more vulnerable to the negative effects of chemotherapies through an increased rate of transcriptional errors and the consequent disruption of the circular and covalently closed mtDNA [31].

The mitochondrial function is modulated by various factors, such as mtDNA, nDNA, nuclear and cellular signaling molecules, mitochondrial reactive oxygen species (mtROS), and ATP formed in other organelles [32,33]. It was demonstrated that chemotherapy such as doxorubicin-an anthracycline used for leukemia and prostate, ovarian, lung, and breast cancer treatment-increases ROS production as an outcome of the redox cycling process [34,35]. Further, chemotherapy can increase the content of O_2_^−^ and H_2_O_2_; these molecules constitute the mtROS, and increase oxidative pressure if not neutralized by endogenous antioxidants [36,37]. Single-stranded DNA damaged by chemotherapies stimulates the activation of enzymes to repair the damage, although this is harmful to ATP stores [38]. NAD^+^ is also a key mitochondrial substrate that impacts many metabolic pathways, such as glycolysis and the tricarboxylic acid cycle [39]. Skeletal muscle is a highly metabolic organ that needs adequate ATP generation; therefore, reduced ATP generation ultimately leads to skeletal muscle dysfunction [40].

Another type of chemotherapy, platinum-derived alkylating agents, causes similar symptoms of skeletal muscle fatigue but through different mechanisms. This is especially the case for oxaliplatin, which was demonstrated to cause dysregulated mitochondrial and energy homeostasis when used to treat colorectal cancer. Its potential mechanism is the induction of mutations in mtDNA or nuclear-encoded mitochondrial proteins, which are beneficial by stimulating the cell death pathways of cancer cells but also lead to mitochondrial dysfunction and the disruption of energy homeostasis in non-cancerous cells, especially in highly metabolic organs such as skeletal muscle [41]. It was proposed that chemotherapy drugs such as oxaliplatin competitively substitute copper (Cu^2^^+^) on copper transporter 1 (CT1), thus reducing the transportation of Cu^2^^+^ and leading to a decrease in the mitochondrial Cu^2^^+^ pool, which is critical for complex IV and COX17 function, as well as oxidative phosphorylation [42]. Moreover, chemotherapies such as the anti-metabolite and topoisomerase inhibitor families regularly induce an increase in mtROS generation and a decrease in mitochondrial pool viability [28]. Excessive ROS generation and impaired mitochondrial respiratory proteins can result in a destructive feedback cycle, leading to mtROS production, respiratory chain defects, mtDNA deletion, and macroautophagy in skeletal muscle (mitophagy) [43]. Therefore, reducing mtROS generation seems to be a potential intervention strategy and would be conducive to the attenuation of muscular atrophy, mitophagy, necrosis, and apoptosis signaling pathways [44,45].

**Figure 2:**
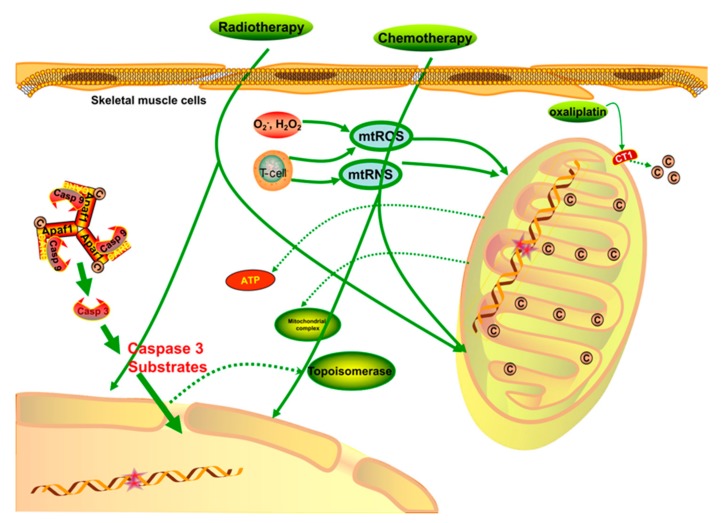
When treated with chemotherapy or radiotherapy, the regular structure and function of mitochondria are damaged through different signaling pathways. The processes involved in the transcription of nDNA and mtDNA are significantly destroyed, and the levels of mtROS and mtRNS are upregulated when skeletal muscle is nonspecifically targeted by chemotherapies. Meanwhile, the respiratory function of mitochondria is weakened by the impaired mitochondrial membrane. The Cu^2+^ capacity is critical for mitochondrial complexes and ATP generation, so when Cu^2+^ is competitively inhibited by some chemotherapies, such as oxaliplatin, the outflow of Cu^2+^ increases, which is harmful to the mitochondrial energy generation. In general, direct chemo/radio-therapy injuries, hyperoxidative stress, and a low energy supply are likely to cause physical fatigue via apoptosis or other detrimental signaling pathways.

Mitochondrial dysfunction plays a major role in the development of diseases associated with energy metabolism, and the impaired energy production or longitudinal depletion of ATP induces increased physical disability, such as that observed in CRF or chronic fatigue syndrome (CFS) [46,47]. The mitochondrial abnormalities associated with these disorders include the loss of mitochondrial membrane integrity, oxidative corruption of translocator proteins [48,49], abnormal muscle mitochondrial morphology, and defective aerobic metabolism, which are different from muscle disuse [50] and oxidative phosphorylation in the striated muscle [51,52]. Some cachexia diseases involve certain T cell disorders, including persistent mitochondrial membrane hyperpolarization and increased ROS and reactive nitrogen species (RNS) synthesis, along with decreased levels of glutathione and ATP [53,54]. The release of disease-associated molecular patterns (DAMPs) into circulation as a consequence of necrosis can lead to systemic inflammation, which in turn amplifies mitochondrial dysfunction in a vicious feedforward loop [47,55]. The possible mechanisms of mitochondrial dysfunction, together with chronic inflammation and oxidative stress or elevated levels of ROS and RNS, cause the destruction of proteins, DNA, and lipid membranes [47]. Nitric oxide (NO) and peroxynitrite can markedly reduce the generation of ATP by inhibiting or inactivating crucial enzymes; meanwhile, the inhibition of the electron transport chain (ETC) increase the formation of ROS, leading to more severe impairment of mitochondrial function [37,56]. Studies have shown that decreased energy production and increased lactate production, as well as the impairment of ATP synthesis, are connected with physiological fatigue, indicating that muscular disorders are likely to play a critical role in CRF [57,58,59]. It has been demonstrated that the origin of severe fatigue lies in mitochondrial respiratory dysfunction and impaired generation of ATP, but the deeper and more accurate picture of the molecular mechanisms that underlie mitochondrial dysfunction and CRF remains unclear [47] (Figure 2).

## 4. Peripheral Immune Activation and Inflammation Dysfunction

CRF is one of multiple concurrent symptoms associated with cancer itself and cancer treatment; however, the interrelations between these symptoms and the underlying mechanisms remain unknown [60,61]. As a symptom of cancer cachexia, CRF is induced by various mediating factors, such as cytokines (tumor necrosis factor α (TNF-α), interleukin 1 (IL-1), IL-6, and interferon-γ (INF-γ)), hormones (insulin, glucagon, leptin, ghrelin, and adiponectin), and neuropeptide-Y [62]. It has been reported that hormones can regulate body weight by adjusting food intake and energy metabolism [63,64,65]. Similar to cachexia, CRF is characterized by systemic inflammation dysfunction, which disturbs the protein and energy balance. For certain neuro-inflammatory, autoimmune, and inflammatory disorders, severe fatigue is related to peripheral immune responses and inflammation due to elevated levels of pro-inflammatory cytokines [66,67]. A variety of cytokines are correlated with the prevalence of sickness behaviors, which are proposed to be the mechanisms of symptom clusters [68,69]. Cytokines are proteins that are generated and/or released by body cells (especially immune cells, such as T cells) that are not only peripheral but also throughout the whole body. Their role is to regulate immune responses through specific receptors [70]. The activation of NF-κB and the subsequent synthesis of pro-inflammatory cytokines are induced by the myeloid differentiation primary response gene (88) (MYD88), which is a general adapter protein used by almost all Toll-like receptors (TLRs) in diverse pathways [71,72,73]. Pro-inflammatory cytokines, such as IL-1, IL-6 and TNF-α act as cell-to-cell mediators in response to external immunologic stressors [74]. However, anti-inflammatory cytokines, such as IL-10, IL-19 and IL-20, are able to regulate the generation and activation of pro-inflammatory cytokines [75]. These pro/anti-inflammatory systems facilitate the body’s return to homeostasis through an inflammatory process by regulating autocrine and paracrine communications in immune cells, as well as immunocytes and other peripheral cells [76].

Fatigue is a common symptom for individuals living with chronic or acute diseases, such as rheumatologic diseases, autoimmune type 1 diabetes, inflammatory bowel diseases, systemic autoimmune diseases, cancers, and infections [67,77,78]. Therefore, in terms of peripheral immune activation or inflammatory dysfunction, CRF may have a similar pathogenesis to other types of pathogenic fatigue. There are variety of factors that contribute to fatigue, such as illness-related characteristics (pain, inflammation, joint damage), physical functions (sleep disturbance and disability), emotional impairment (depression and anxiety), and personal conditions (gender, work, social relationship, education, and whether the patient has a partner a partner) [67,79]. Some research has reported that the prevalence and severity of fatigue are associated with the serum levels of inflammatory cytokines, such as IL-6, TNF-α, IL-1 receptor antagonist (IL-1RA), and especially IL-8, a significant factor of pain and fatigue in lung cancer patients [80,81]. Upregulation of IL-6 and NF-κB has also been reported in cancer patients with sleep disturbances [82]. Furthermore, chemotherapy is one of the CRF-inducing factors because it triggers the generation and secretion of inflammatory cytokines by tumor or immune cells [83]. The increased levels of TNF-α and other cytokines in the periphery are significant predictors of the development of the active disease and fatigue severity that affect the majority of patients with cancer or those receiving cancer treatments [84,85]. The sense of fatigue can persist for over 10 years after chemotherapy treatment, meanwhile, pro-inflammatory cytokine levels remain high, and affect central nervous system (CNS) functions and other behavioral symptoms [86,87]. Moreover, high levels of fatigue have been reported among HIV/AIDS patients undergoing immunotherapies, and their plasma IFN-γ, IL-2, and TNF-α levels were determined to be lowered by antiretroviral therapy, suggesting that there is an intimate relationship between changes in inflammatory cytokines and the degree of fatigue [88].

It is generally accepted that fatigue is related to the loss of muscle mass or altered mood, but research has found that phenotypic fatigue and depressed mood behaviors, such as decreased voluntary wheel-running activity, resignation, and anhedonia, are not correlated with the contractile function of skeletal muscle, indicating that fatigue is likely associated with behavioral activity rather than muscle dysfunction [89]. After injecting murine models with IL-1, their social exploration ability and body weight decreased, and hypersomnia increased. These phenomena were improved by the administration of anti-inflammatory agents, such as IL-1RA or IL-10 [90,91]. Furthermore, in research on military personnel with insomnia, there was a higher degree of CRF in the restorative sleep group than the persistent insomnia group [92]. A therapeutic study showed that when pro-inflammatory cytokines were reduced by biological agents, fatigue severity was reduced accordingly, indicating that inflammatory disorders might be factors that induce or exacerbate CRF [93]. Additionally, fatigue has been found to co-exist with inflammation-associated anemia that was caused by a decrease in iron levels due to thyroid insufficiency or impaired HPA axis function mediated by IL-6 [94,95].

**Figure 3:**
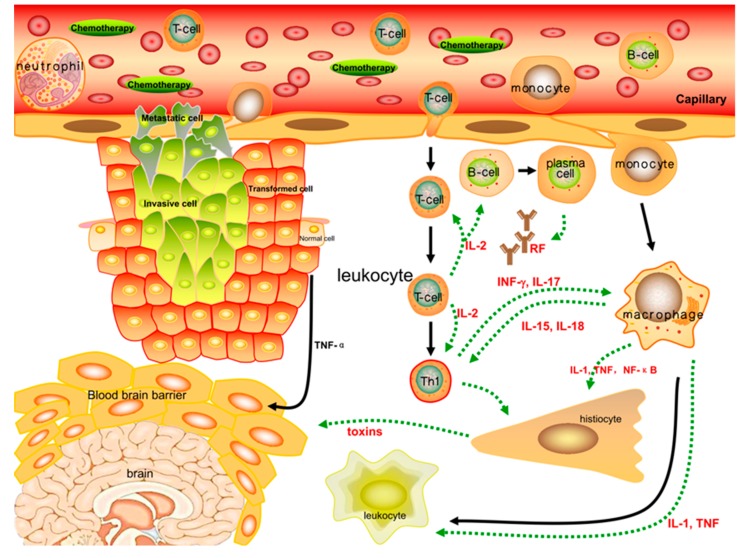
Inflammatory cytokines such as TNF-α, IL-1β, IL-2, IL-6, and INF-γ, which are correlated with the severity of fatigue, play a significant role in peripheral immune activation. Cytokines are primarily generated by immune cells and regulate inflammatory responses through peripheral, neural, and even systematic circulations. The pro/anti-inflammatory function encourages the body to maintain relative homeostasis by autocrine and paracrine communication between immune cells. When stimulated by chemotherapies, the inflammatory responses of immune cells are further strengthened, and the secretion of cytokines such as NF-κB, IL-1β, and TNF-α into the peripheral or neural circulation is increased. These changes lead to more severe fatigue symptoms that are closely linked to the incidence of CRF.

Indeed, early stimulators of chronic inflammation include pro-inflammatory cytokines and other molecules synthesized in response to pathogen invasion, as well as NF-κB activated by macrophages and other sentinel cells [96,97]. Cytokines, ROS, and RNS upregulated by NF-κB tend to maintain and amplify inflammatory and immune responses in a TLR-radical cycle in a feed-forward manner [37,98,99,100]. Moreover, pro-inflammatory cytokines can amplify the function of NF-κB through canonical pathways and lead to mutually elevated activity [101,102]. ROS and RNS are harmful to lipids, proteins, and DNA and lead to the formation of redox-derived DAMPs [103,104]. In return, these redox-derived DAMPs, along with TLRs, facilitate the genesis of NF-κB, cytokines, ROS, and RNS [105]. Briefly, the engagement of TLRs mediated by DAMPs can maintain and amplify chronic inflammation and immune activation [37,73]. Furthermore, elevated levels of NF-κB, pro-inflammatory cytokines, ROS and RNS tend to disrupt epithelial tight junctions in the intestine, resulting in the release of gram-negative bacteria containing lipopolysaccharides (LPS) into the circulation system. As a pathogen-associated molecular pattern (PAMP), this can amplify the TLR-radical cycle [66]. Thus, intestinal permeability is further altered by the translocation of LPS (from gut bacteria), which interacts with TLRs. This is a potential cause of chronic immune activation, which can lead to major depression, CRF, neuro-inflammatory disorders, and other autoimmune diseases [37,56,106]. In other words, the TLR-radical cycle, which leads to increased levels of pro-inflammatory cytokines and ROS or RNS, is likely to be a potential mechanism that drives the development of severe fatigue in cancer patients [73] (Figure 3).

## 5. Neuron and Central Nervous System (CNS) Disorder

The CNS is a significant factor in the induction of CRF by sensing inflammation, integrating signals from the peripheral nervous system, and stimulating downstream alterations in body mass and systemic metabolism [107]. Fatigue is also one of the characteristics of major depression induced by chronic systemic inflammation and the activation of neuroglia cells [108,109,110,111]. It is generally believed that CRF is related to peripheral mechanisms, such as skeletal muscular metabolism, and pro-/anti-inflammatory cytokines. However, a study investigating the mechanisms of neuromuscular fatigue in children and adults found that children experienced more severe central fatigue than adults, which could be instructive in developing a therapeutic strategy that limit the recruitment of motor units to avoid extensive peripheral fatigue [112]. Lower peripheral fatigue can be transmitted to the CNS via feedback mechanisms and be translated to reduced central fatigue, indicating that fatigue is highly related to CNS dysregulation [113]. In pro-inflammatory stress, signals integrated by the hypothalamus from peripheral systems are translated into neuroendocrine dysregulation, neural signaling alteration, and systematic metabolic derangements. Peripheral pro-inflammatory cytokines induced by systemic inflammatory responses enter the brain by various routes to activate microglia and astrocytes and trigger the production of cytokines and other neurotoxins, which lead to neuroinflammation in the CNS [56,114]. Research has demonstrated that the intracerebroventricular (ICV) injection of pro-inflammatory cytokines stimulates anorexia, lethargy, and catabolism at lower doses of peripheral injection. In addition, the attenuated dynamic regulation of the vascular access and blood-brain-barrier (BBB) function induce higher sensitivity of specifical brain areas to metabolic and inflammatory signaling molecules [115,116,117,118,119]. Inflammatory cytokines secreted by tumor cells can enter the brain through the damaged BBB and trigger neuroinflammatory responses, which leads to severe fatigue symptoms that are unlikely to be alleviated by regular physical movement.

The mediobasal hypothalamus (MBH) contains several neuronal peptides, including proopiomelanocortin (POMC), and cocaine-and-amphetamine-regulated transcript (CART) [120]. The POMC is a precursor polypeptide that can synthesize α-melanocyte-stimulating hormone (αMSH), adrenocorticotropic hormone (ACTH), enkephalin, and β-endorphine (β-EP) through several pathways [121]. The αMSH in the brain can decrease appetite and energy storage. CART is a neuropeptide that can increase locomotor activity and decrease food intake and act as an endogenous psychostimulant [122]. Agouti-related peptide (AgRP) is a significant orexigenic peptide that is characterized as an antagonist of melanocortin receptor 4 (MCR4) and an inhibitor of POMC neuron activity, thus inducing increased appetite and energy storage [123,124]. Neuropeptide Y (NPY) is another orexigenic peptide that has been proposed to be an effective orexigenic factor in the brain and autonomic nervous system, similar to intestinal peptide YY (PYY) [125,126]. These substances are considered to be closely related to the onset of CRF. It has been proposed that the MBH is closely related to CRF by receiving central and peripheral inflammatory signals and providing primary neuronal substrates that link inflammation to fatigue, anorexia, skeletal muscle catabolism dysfunction, and other diseases [127,128,129]. Furthermore, researches have shown that catabolic pathways in the muscle that are related to CNS regulation and muscle are the main targets of pathologic tissue impairment in the disease. This observation indicates that the catabolism of skeletal muscle is one of the factors of cachexia [130,131]. The cytokines entering the CNS stimulate the HPA axis function to promote catabolism of carbohydrates, proteins, and lipids in peripheral tissue, including skeletal muscle and adipose tissue [132].

Studies have indicated that peripheral fatigue results from the depletion of energy stores, synthesis of side-products, and decrease in muscle contractile capacity, which are related to immunological and genetic responses [133,134,135]. The locomotor system is regulated by neuronal networks of central pattern generators (CPGs) in the spinal cord. These are special groups of neurons that produce basic rhythmic patterns [136]. Some specific regions in the brain, such as the prefrontal cortex, the motor cortex, cerebellum, and basal ganglia, are essential for locomotor function and voluntary rhythmic motor patterns [137,138]. Moreover, nitric oxide (NO), the cyclic guanosine 3′, 5′- monophosphate pathway and soluble guanylyl cyclase regulate locomotor activity through NO-dependent or NO/cGMP/KATP pathways, which was demonstrated in anti-nociception in rats [139]. Increased levels of nitrite in both the plasma and brain of resistance-exercised rat models were found in an experimental study [140]. Intracerebral monoamines/neurotransmitters, such as serotonin (5-HT), dopamine (DA), and noradrenaline (NA), have been demonstrated to be associated with central fatigue [141]. 5-HT regulates neurotransmission and various physiological functions related to central fatigue and locomotor activities [142]. Higher 5-HT concentrations in the brain are stimulated by increased tryptophan levels, and can also facilitate 5-HT’s crossing of the BBB, which affects locomotor performance [138]. Meanwhile, increased DA in the brain induces blood calcium influx into the CNS through the proposed mechanism of calcium/calmodulin-dependent DA synthesis and upregulation of DA receptors [143]. Additionally, the noradrenergic neurons that synthesize NA by the hydroxylation of DA can regulate movement through the innervation of the cerebral cortex, subcortical areas, cerebellum, and the brain stem [144]. Nevertheless, the neurotransmitter regulation, synthesis, and secretion functions of the CNS can be influenced by muscle metabolism byproducts, such as ammonia, a metabolic end-product from gastrointestinal tracts [145].

**Figure 4:**
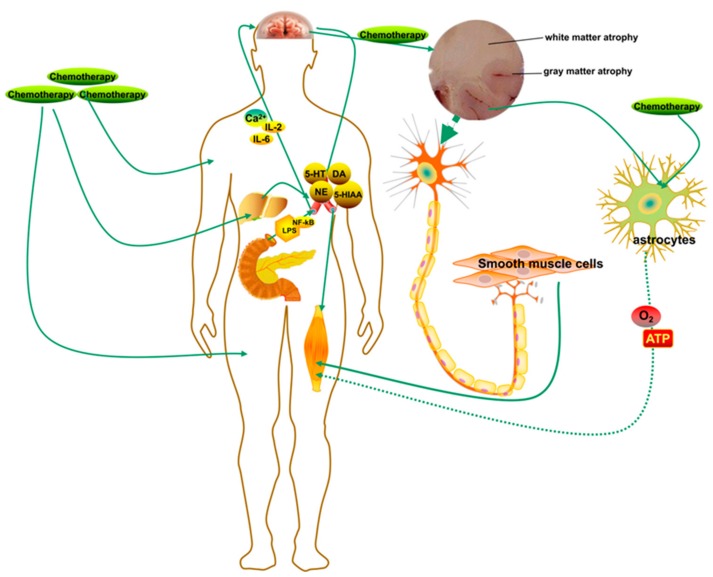
The central nervous system (CNS) integrates the signals from peripheral circulations to inhibit or amplify immune signals through neural regulation mechanisms. On the account of inflammatory stress, peripheral inflammatory cytokines enter the brain through various routes to activate microglia and astrocytes and to generate neurotoxins that cause neuroinflammation in the CNS. Furthermore, inflammation of the nervous system leads to severe disorders of systemic circulations, such as decreased blood-brain barrier (BBB) strength, atrophy of spinal gray matter, or decreased muscular innervation. A nervous system affected by peripheral or central inflammatory responses is inclined to destroy muscle cells and inhibit the generation of energy and nutrition, leading to motor unit decrease and severe fatigue, both physical and mental.

There is acceptance of the positive association between changes in brain activity and cognitive fatigue, and these connections typically occur in several areas of the brain, such as the basal ganglia and prefrontal cortex [146,147,148]. One hypothesis is that the stimulation of fatigue in neurological diseases is caused by the inordinate signal integration processed in the basal ganglia, which regulate inputs from the limbic system and outputs to the motor cortex, and, potentially, pathological states in the white and gray matter [149,150,151]. Gray matter atrophy occurs in some early diseases, such as clinically isolated syndrome (CIS), and the extent of gray matter atrophy correlates significantly with the degree of cognitive impairment and physical disability [152,153,154]. In fatigue, a clear relationship has been demonstrated to exit between global or localized gray matter atrophy and hypoperfusion [155,156]. Moreover, other research has found an association between fatigue and glucose hypometabolism in the frontal cortex and basal ganglia and a decreased N-acetyl asparagine/creatine ratio in basal ganglia [157]. Glial cells, such as astrocytes and protoplasmic astrocytes, play crucial roles in neurometabolic and neurovascular coupling and the delivery of oxygen and energy to central neurons [158,159,160]. It was reported that astrocytes form the vast bulk of gray matter, and the loss of gray matter in the early development of many diseases is due to the loss of astrocytes, which correlates significantly with the severity of inflammation [161]. These findings could explain the basal ganglion dysfunction, which leads to changes in brain activity and the progression of cognitive fatigue that correlates with CRF (Figure 4).

## 6. Conclusion and Discussion

CRF is frequently experienced by patients after receiving cancer treatment, including chemotherapy or radiotherapy, and the condition persists for a long time. It is often emphasized that the early natural history of CRF has not been systematically researched, so its symptoms, mechanisms, and prevention or treatment strategies are not entirely understood [6,162]. Various clinical studies have indicated that potential mechanisms of CRF lie in skeletal muscle metabolism, as well as pro- and anti- inflammatory functions. Moreover, neural or CNS dysfunction are the primary factors that induce the occurrence or persistence of CRF. However, there seem to be no treatment-related factors that are concordant with systematic reviews that could predict the incidence or treatment of CRF [163]. Recent research has found that, in the context of sickness such as cachexia, peripheral leukocytes concentrate in the brain and significantly affect the neuroinflammatory response. Meanwhile, research on cancer immunology has shown that leukocytes also significantly contribute to the inflammatory immune response in the brain [107,163]. Furthermore, a growing body of evidence indicates that the hypothalamus plays a driving role in the pathophysiology of the acute or chronic illness response. When stimulated by inflammatory cytokines, the hypothalamus stimulates skeletal muscle catabolism and lipolysis by regulating neurotransmitters, systemic circulation, and autocrine and paracrine signaling. However, the method by which cancer treatments such as chemotherapy and radiotherapy trigger the occurrence of CRF remains unknown, despite the increasing amount of research that indicates that skeletal metabolism, the inflammatory response, and CNS function are potential mechanisms of CRF [107]. Furthermore, rodent models are imperative to providing insight into the underlying stimulating molecular factors of CRF.

It is proven that the inflammation disorder drives the pathology in early stages of the disease, and a significant role in the progress of the disease is played by mitochondrial dysfunction, induced by oxidative damage, which leads to the reduced activity of mtDNA and mitochondrial complexes [164]. However, there is a great variability in patients’ cytokine levels and degree of fatigue during their treatment trajectories [165]. The hypothesis that genetic differences in pro-inflammatory cytokines could influence this phenomenon is currently accepted. Although a Genetic Risk Index was developed to predict a high risk of fatigue in women, there is no systemic explanation of the variability in CRF, and additional research is needed to identify CRF genetic variants [165,166]. 

CRF is characterized by a subjective feeling of severe fatigue, which is greater than general fatigue that would be expected under a special condition. However, the accurate pathophysiology of CRF has not been clearly explained, and whether CRF is peripheral or central or both has never been clarified [167,168]. Generally, fatigue can occur from brain cells to skeletal muscle basic contractile units. Central fatigue refers to the lack of motivation, central nervous system transmission or recruitment changes, and cognitive disorder or depression. However, peripheral fatigue includes decreased contact transfer, muscle point activity, and muscle contraction activity. To now, the paramount question then becomes to what extent can peripheral or central fatigue explain the pathogenesis of CRF. From a developmental perspective, a well understanding of peripheral or central fatigue can provide a pathological correlation that is lacking in the current cognition of CRF. Production of pro-inflammatory cytokines, oxidative stress, and muscle tissue injury occur from cancer or its treatment and promote a systemic effect some distance from the origin of cancer. On the other hand, many patients with breast cancer receiving chemotherapy present a chemo-brain phenomenon, which can be manifested as depression or impaired cognition [169]. This is an interesting phenomenon, suggesting that we ought to explore the pathogenesis of CRF from the perspective of the intrinsic relationship between peripheral and central fatigue, which is based on the sufficient understanding of peripheral and central fatigue. Inflammatory cytokine release, oxidative stress, and mitochondrial energy supply disorders can all occur in the peripheral and central system, which seem to be responsible for CRF, but the intrinsic relationship between muscle fatigue and neuromuscular fatigue in unknown.

In summary, systemic clinical and animal research on the CRF is essential to identify the triggering factors and mechanisms of CRF via histopathological, molecular biological, and biochemical methods. Clarifying the changes in the microstructure of the skeletal muscle, mitochondrial membrane, and brain tissue would be conducive to understanding the physiological mechanisms of CRF. Furthermore, a comparison between patients with different kinds of cancer, therapeutic schedules, and lifestyles would contribute to finding common factors of the mechanisms of CRF. Current research indicates that serum eotaxin, IL-1RA, monocyte chemoattractant protein 1 (MCP-1), and neurocognitive performance are inversely associated with fatigue before chemotherapy; however, serum MCP-1, macrophage inflammatory protein (MIP)-1a, and daily energy expenditure were significantly associated with fatigue after chemotherapy. Furthermore, only the serum IL-12 has been correlated with fatigue both before and after chemotherapy [170]. Hence, animal models should be developed to explore the exact neural and molecular pathways of these phenomena [171]. Creating accurate animal models that mimic the pathological features of CRF should be a priority for exploring the exact mechanisms of CRF before and after cancer treatment. Furthermore, exploring the intrinsic relationship between peripheral and central fatigue may also contribute to a better understanding of the pathogenesis of CRF. In summary, skeletal muscular and mitochondrial dysfunction, pro/anti-inflammatory cytokines (both peripheral and neural), and CNS disorders-especially hypothalamus function-are promising research directions to further the current understanding of the pathogenesis and clinical intervention strategies of CRF.
